# Ticagrelor Can Be an Important Agent in the Treatment of Severe COVID-19 Patients with Myocardial Infarction

**DOI:** 10.4274/balkanmedj.galenos.2020.2020.4.100

**Published:** 2020-06-01

**Authors:** Ercan Akşit, Bahadır Kırılmaz, Emine Gazi, Fatih Aydın

**Affiliations:** 1 Department of Cardiology, Çanakkale Onsekiz Mart University School of Medicine, Çanakkale, Turkey; 2 Clinic of Cardiology, Eskisehir City Hospital, Eskişehir, Turkey

To the Editor,

In cases of pneumonia developing due to coronavirus disease 2019 (COVID-19), it has been shown that 71.4% of non-survivors and 0.6% of survivors had disseminated intravascular coagulation (DIC), and the majority of non-survivors had increased D-dimer level (1). TNF-α and IL-6 variants are also known to be risk factors for pneumonia-induced septic shock in intensive care patients (2). We believe that the ADP receptor inhibitor, ticagrelor, should be considered in the treatment of concomitant COVID-19 pneumonia and myocardial infarction (MI) because it may contribute to patient survival. There are three main reasons for this, considering the pathogenesis of COVID-19 pneumonia and the course of the disease. First, based on the subgroup analysis of the PLATO study, the initially identified pleiotropic effects of ticagrelor were evident such that sepsis and pulmonary infections were less common in individuals using ticagrelor. This is because it reduces levels of proinflammatory factors as well as platelet reactivation via A2A and A2B adenosine receptors and ultimately prevents DIC development (3). Second, ticagrelor has been shown to reduce lung injury by reducing thromboinflammatory markers in patients with pneumonia (4). Finally, patients with COVID-19 may develop superinfections during the course of their treatment. Such additional problems may not be detected in patients who are already treated under challenging conditions. A recent study showed that ticagrelor at a conventional dose has shown a higher antibacterial activity against many antibiotic-resistant gram-positive bacteria compared to the most potent antibacterial agents currently available (5).

In conclusion, COVID-19 pneumonia is a pandemic with significant mortality that is spreading very quickly, and there is uncertainty regarding when it will be completely terminated, and more importantly, no clear treatment has been yet confirmed (1). Its coexistence with MI, which is the most fatal disease in the world, makes the situation even more devastating. We believe that ticagrelor will play a very important role in the survival of patients, especially those with concomitant COVID-19 pneumonia and MI. Considering the pleiotropic effects of ticagrelor, it can significantly contribute to the survival of patients with MI coexisting with COVID-19 regardless of whether percutaneous intervention is introduced or medical treatment is given. Even in cases where COVID-19 is not accompanied by MI, the use of ticagrelor should be considered in patients whose prognosis worsens and D-dimer level gradually increases.
